# Microalgae as Contributors to Produce Biopolymers

**DOI:** 10.3390/md19080466

**Published:** 2021-08-19

**Authors:** Rozita Madadi, Hamid Maljaee, Luísa S. Serafim, Sónia P. M. Ventura

**Affiliations:** 1 Department of Agricultural Biotechnology, University College of Agriculture and Natural Resources, University of Tehran, Karaj 77871-31587, Iran; rozamadadi@gmail.com; 2 CICECO—Aveiro Institute of Materials, University of Aveiro, Campus Universitário de Santiago, 3810-193 Aveiro, Portugal; h.maljaee@ua.pt (H.M.); luisa.serafim@ua.pt (L.S.S.); 3 Chemistry Department, University of Aveiro, Campus Universitário de Santiago, 3810-193 Aveiro, Portugal

**Keywords:** microalgae, bioplastic, starch, protein, polyhydroxyalkanoates, biopolymer blends

## Abstract

Biopolymers are very favorable materials produced by living organisms, with interesting properties such as biodegradability, renewability, and biocompatibility. Biopolymers have been recently considered to compete with fossil-based polymeric materials, which rase several environmental concerns. Biobased plastics are receiving growing interest for many applications including electronics, medical devices, food packaging, and energy. Biopolymers can be produced from biological sources such as plants, animals, agricultural wastes, and microbes. Studies suggest that microalgae and cyanobacteria are two of the promising sources of polyhydroxyalkanoates (PHAs), cellulose, carbohydrates (particularly starch), and proteins, as the major components of microalgae (and of certain cyanobacteria) for producing bioplastics. This review aims to summarize the potential of microalgal PHAs, polysaccharides, and proteins for bioplastic production. The findings of this review give insight into current knowledge and future direction in microalgal-based bioplastic production considering a circular economy approach. The current review is divided into three main topics, namely (i) the analysis of the main types and properties of bioplastic monomers, blends, and composites; (ii) the cultivation process to optimize the microalgae growth and accumulation of important biobased compounds to produce bioplastics; and (iii) a critical analysis of the future perspectives on the field.

## 1. Introduction

The circular economy model (CEM) to overcome the current problems associated with the growth in consumption and production demands has received much attention. This model is mainly based on the resource, recovery, and recycle strategy in order to limit the consumption of raw materials and natural resources [1]. CEM successfully enables a concurrent assessment of social, economic, and environmental concerns, which is missing in the previous models [2]. Recently, the circular economy has relied on the biorefinery concept involving biomass and renewable resources, aiming to minimize greenhouse gas emissions and waste disposals [3]. Biorefinery plays a key role in moving towards a net-zero society [4]. Annually, more than 400 million tons of plastic are produced worldwide, about one-third of which ends up in landfills, freshwater lakes, rivers, and oceans as plastic waste [5]. Despite the variety of applications of petroleum-based plastics and petrochemical-based polymers, they are non-biodegradable and can cause numerous problems in the whole ecosystem [6]. A rapid increase in the production of synthetic plastics has been associated with considerable energy consumption and GHG emissions along with the release of hazardous chemicals [7]. Over the years, many researchers have attempted to find environmentally friendly and sustainable resources as an alternative for plastic production [7]. Bioplastics are recognized as promising materials over conventional plastics and can be produced from renewable biomass sources, agricultural byproducts, and microorganism sources. Their production essentially needs a low energy consumption in comparison to petroleum-based conventional plastics [7]. The first generation of biopolymers, which are produced from the raw feedstock, require arable land, nutrients, and fresh water, eventually competing with food production. This problem can be overcome with the use of agricultural wastes. However, these resources are limited and insufficient for bioplastic production. For this reason, bioplastics produced from fast-growing microorganisms such as bacteria and microalgae have attracted increased attention. Besides the high potential of microalgae in mitigating CO_2_, their cultivation requires less water than land crops, and unlike food crops, algae are not used as a primary food source for human beings, meaning that they can be used as an excellent source of bioplastic production while having less impact on food security [8].

Microalgae (and some cyanobacteria) are capable of producing great amounts of lipids, proteins, and carbohydrates, which are the most significant substances in the main composition of products of biobased origin such as bioplastics, biopolymers, and biobased polyurethane [9,10]. In the most recent years, microalgal biomass, used either as biomass directly or as a feedstock for secondary processes, has been noticed as a potential source of materials improving several fields, including bioplastic production [11].

**Figure 1:**
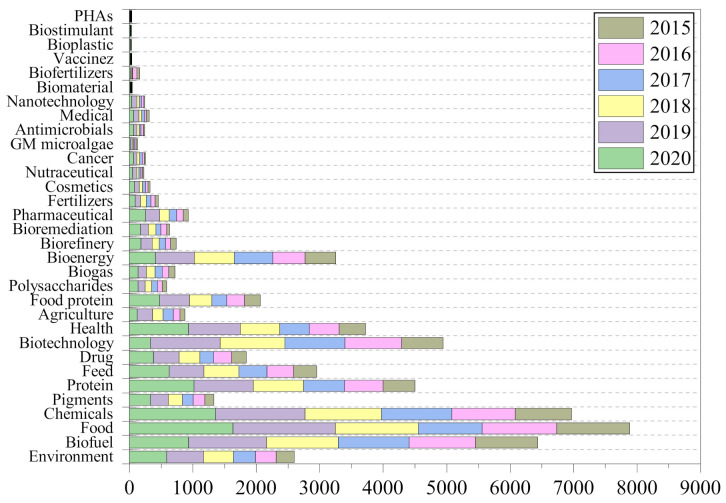
Main research domains identified in the microalgae scientific production in the world (from Web of Science in March 2021).

Searching in the literature using Web of Science (March 2021), from 2015 up to now, 32 research areas were identified regarding “microalgae”, as depicted in Figure 1. The biggest incidence in the literature related to microalgae was on the topic “food” with 8104 publications, followed by “chemicals” with 7164 publications and “biofuels” with 6586 publications. These keywords cover very broad topics; however, more specific markets seem to be neglected so far, since these correspond only to a few publications. As an example, many articles have been published in the fields of “proteins for food” (2123 publications), “pigments” (1400 publications), and “biogas” (747 publications). Research about the positioning of microalgae as feedstocks under the concept of biorefinery and circular economy has increased from 2015 to 2021; however, much more needs to be explored to develop the process platforms able to give a sustainable solution to the circular economy and biorefinery demands.

**Figure 2:**
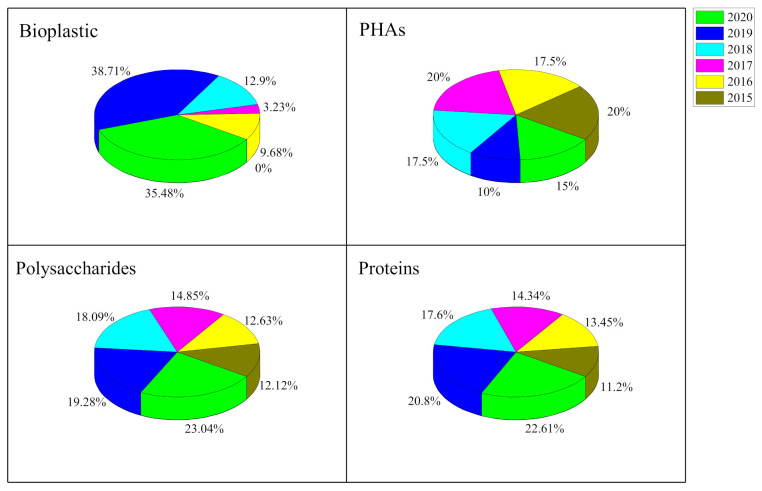
The percentage of scientific publications about microalgae applications in the fields of bioplastics, PHAs, polysaccharides, and proteins.

Chemicals from algae include biopolymers, bioplastics, biofuels, biolubricants, dyes, solvents, agrochemicals, epoxies, acids, commodity chemicals, aldehydes, defoamers, food additives, and inks [12]. The ability of microalgae to grow in nonarable land at an acceptable growth rate and high photosynthetic conversion efficiency makes them a suitable feedstock for bioplastic production [8,11]. Microalgae have been recognized as a sustainable resource of biomass to be applied as biofertilizers and in human and animal nutrition, wastewater treatment, nitrogen fixing, CO_2_ mitigation, and bioenergy production [4]; they also contain several bioactive compounds with applications as stable-isotope biochemicals, nutraceuticals, and cosmetics, just to mention a few. To be more precise, a literature search devoted to the principal markets using “microalgae” or microalgae-based compounds was also contemplated. In general, the outputs obtained were focused on “biomaterials” (46 publications), “vaccines” (44 publications), “polyhydroxyalkanoates (PHAs)” (44 publications), “biostimulants” (35 publications), and “bioplastics” (33 publications). Figure 2 shows the percentage of scientific publications since 2015 reporting microalgae as contributors to the production of bioplastics, PHAs, proteins, and polysaccharides per year (searched in Web of Science in March 2021). More precisely, and according to Figure 2, in the last two years, the application of microalgae in “PHA” production has been poorly explored when compared with other topics. Nevertheless, the number of publications focusing on the use of microalgae in the production of bioplastics has increased, which may be attributed to the fact that algae are starting to be considered as efficient and economically viable biorefinery feedstocks. The commercial field of microalgae is indeed showing its economic importance. An example is the price of products such as lactic acid, butanol, and PHAs, reaching values of around USD 1300–7000 per ton [10].

Despite the increased relevance of microalgae and their bioactive compounds in several markets, much more needs to be done considering microalgae production, namely in what concerns the understanding behind the maximization of cell production and the maximization of the production of the most relevant compounds in order to increase processability and market competitiveness, besides reducing the production costs [13]. 

This review aims to provide a comprehensive analysis of the potential of microalgal biopolymers including PHAs, proteins, cellulose, and starch in the production of biobased plastics, blends, and composites. While the species used as resources for bioplastic production, the microalgae cultivation methods, and the bioplastic material production methods were reviewed last year in [14], in this work, our intention is to complement the analysis and discussion around the subject. Thus, this review begins by overviewing the main types and properties of bioplastic monomers and blends, slightly crossing information on properties with the optimization of the cultivation conditions to best accumulate these biobased compounds, and this is followed by the most recent approaches on the extraction of biopolymers from microalgae. Furthermore, this review ends with a critical analysis of the future perspectives on the field, under the strategy of the circular economy.

## 2. Polyhydroxyalkanoates (PHAs) in Microalgae

**Figure 3:**
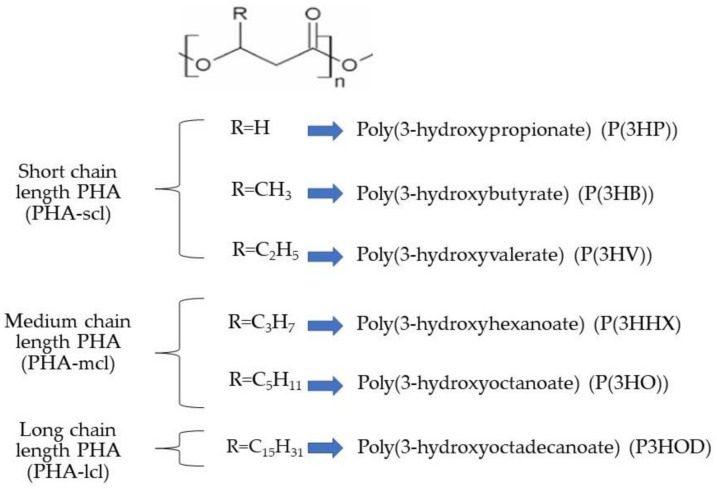
Chemical structure of PHAs and their classification based on the associated R-group, reproduced from [17,22].

PHAs are polyesters of hydroxyalkanoates produced by bacterial and algal cells, as an intracellular carbon source from sugar and/or lipids [15]. PHAs are considered suitable substitutes for petrochemical-based plastics such as polypropylene in plastic bags and containers considering their similar physical properties [15,16]. PHAs are formed of repeated ester units containing a carbon chain bound to an R-group and two oxygen atoms [17,18] (Figure 3). In terms of properties, they are characterized by a high hydrophobicity, consequently resulting in a low solubility in water. They are considered inert, nontoxic, and indefinitely stable in the air. PHAs can show thermoplastic and elastomeric properties, very high purity within the cell, high resistance to UV light degradation, and low solvent resistance [13] and are biodegradable and biocompatible. These properties make them appropriate materials in various applications, such as surgical devices (bone plate, repair patches, screws, and orthopedic pins), replacements or scaffolds for damaged tissue in tendon repair devices, wound dressing, vein valves, and bone marrow scaffolds [19,20,21]. 

PHA bioplastics are classified into three major subdivisions based on their R-group and composition of the monomer units [18]. The R-group is made up of hydrogen or hydrocarbon chains up to C15 in length. Short-chain-length 3-hydroxyalkanoates (scl-3HA) have 3–5 carbon atoms, medium-chain-length 3-hydroxyalkanoates (mcl-3HA) have 6–14 carbon atoms in the chain, and long-chain-length hydroxyalkanoic acids (lcl-PHA) have more than 15 carbon atoms [13,23]. The mechanical properties of PHAs, including hard crystalline and elastic behavior, can vary from one PHA to another, depending on their composition in monomer units [17]. The monomer composition determines the state of polymers, which can range from rigid and brittle thermoplastics to elastomers and rubbers [13]. The increase in the number of comonomers, i.e., the increase in the chain length of a PHA, allows an increase in its elasticity. Short-chain-length PHAs include poly-3-hydroxypropionate (P(3HP)), poly(3-hydroxybutyrate) (P(3HB)), and poly(3-hydroxyvalerate) (P(3HV)) and are also indicated in Figure 3. These compounds have some common properties, namely negligible solubility in water and high resistance to moisture and hydrolytic degradation. P(3HP) is also known to be highly crystalline, while P3HB, with a crystallinity of around 60%, can be considered as a sustainable alternative to PP. These are characterized by a significant thermoplasticity and resistance to UV light. P(3HB) normally has a melting temperature (*Tm*) of between 171 and 177 °C, a glass transition temperature *(Tg*) of about 2 °C, a tensile strength of between 18 and 43 MPa, and an extension to break of between 3 and 5% [18,24,25]. Thermoplastic processability, hydrophobicity, biodegradability, biocompatibility, and optical purity are the main characteristics of P(3HB) [18,26,27]. Medium-chain-length PHAs, including poly-3-hydroxyoctanoate (P(3HO)) and poly-3-hydroxyhexanoate (P(3HHx)), are more elastomeric than short-chain-length species. These types of PHA are characterized by having lower glass transition and melting temperatures and higher elongation-to-break ratios when compared with scl-PHA [28]. P(3HHx) exhibits different behaviors when the percentage of monomers is varied. Lower percentages of comonomer allow obtaining compounds with a hard state having some elasticity, while high levels of comonomer lead to a soft and rubbery-like PHA [17]. Long-chain-length PHAs, e.g., poly-3-hydroxyoctadecanoate (P3HOD), have crystallinity indexes compared to scl-PHA and mcl-PHA [17]. 

Thanks to the properties of PHAs, researchers and manufacturers intend to utilize these species of biopolymers in packaging, printing inks, coatings, laminations, waxes, binders, and adhesives [27]. Thus, and considering, for example, the high biocompatibility and sustainability associated with P(3HB), the number of companies producing these biopolymers is not surprising, namely the P(3HB) Industrial (Serrana, Brazil), Tianan (Ningbo, China), CJ CheilJedang (Dongho-ro, Korea)), Kaneka (Minato, Japan), and Biomer (Schwalbach am Taunus, Germany) [29]. The copolymer of 3-hydroxybutyrate and 3-hydroxyvalerate, poly(3-hydroxybutyrate-co-3-hydroxyvalerate) (P(3HB-co-3HV)), is known to be more flexible than P(3HB) [22] and, therefore, is much more interesting from the commercial point of view.

*Tm*, *Tg*, crystallinity, tensile strength, extension to break, and molecular mass are the key properties normally used to define the physical behavior of PHAs. In terms of thermal characteristics, PHAs show a melting temperature ranging from 50 to 180 °C and a crystallinity between 30 and 70%, which depends on the polymer composition [30]. PHAs with 60–80% crystallinity behave as a rigid material, conversely; PHAs with 30–40% crystallinity are usually more flexible. Therefore, a lower degree of crystallinity favors the possibility of being industrially applied due to the improved processing characteristics [31]. The commercial suitability of PHAs also depends on their molecular mass and polydispersity. Their molecular mass should be higher than 4 × 10^4^ Da because a lower value leads to poor mechanical properties. PHAs usually present molecular mass between 2 × 10^5^ and 3 × 10^5^ Da [13], which is normally dependent on the extraction method. It is worth noting that the variation of this polymeric property depends on several factors such as the type of microbial species used and the associated growth conditions [32]. Companies in the PHA bioplastic business are widespread in North America, Europe, and Asia-Pacific. Companies such as CJ CheilJedang (Dongho-ro, Korea)., Ecomann Biotechnology Co. Ltd. (Shenzhen, China), Tianjin GreenBio Materials Co. Ltd. (Tianjin, China), Danimer Scientific (Bainbridge, USA), Mango Materials (Albany, USA), Newlight (Huntington Beach, USA), and Biomer (Schwalbach am Taunus, Germany) are considered the main PHA producers [29]. Industrially, PHAs are usually produced in large fermenters by heterotrophic bacteria fed with large amounts of organic carbon sources, which corresponds to 50% of the total production costs [22]. Algae are suitable alternative raw materials for PHA production, which are normally associated with lower costs [13]. Microalgae, as autotrophs, are photosynthetic microorganisms that can convert solar energy and carbon dioxide (CO_2_) into several valuable bioproducts with the O_2_ evolution [27]. Microalgae are categorized into four major taxonomic groups: (i) diatoms (Bacillariophyceae), (ii) green algae (Chlorophyceae), (iii) cyanobacteria or blue-green algae (Cyanophyceae), and (iv) golden algae (Chrysophyceae). Several cyanobacteria, such as *Synechocystis* sp., *Spirulina* sp., *Nostoc* sp., *Oscillatoria* sp., and *Calothrix* sp., were reported to have PHA content of 1–10%, depending on the species [33]. P(3HB), the most common short-chain-length PHA (scl-PHA) produced by cyanobacteria, is one of the most investigated and commercially available [18,26,27]. *Calothrix scytonemicola*, *Spirulina* spp., *Aphanothece* spp., *Gloeothece* spp., and *Synechococcus* spp. are known cyanobacteria species that can produce P(3HB) using CO_2_ as a carbon source [18]. For most organisms, including cyanobacteria, PHAs act as energy and carbon storage compounds [34]. Despite the low content of PHAs in microalgae, still far from the contents obtained with bacteria, the conversion of atmospheric CO_2_ into PHAs without the use of a carbon source, which as mentioned before can account for a significant part of production costs, is very advantageous due to the necessity to compensate for the increasing emissions of CO_2_. Moreover, when compared with processes using bacteria, the development of microalgae processes for the production of PHAs is in a much earlier stage of development, and the optimization of the process is far from complete [35]. Unlike the conventional bioplastics which are from food staples, microalgae are a promising source of bioplastic production thanks to the high content of carbohydrate polymers and protein [36].

As observed for P(3HB) of other origins, P(3HB) produced from algae was demonstrated to have better recyclability, biocompatibility, biodegradability, plasticizing capacity, and usability than petroleum-based plastics [37,38]. Das et al. [38] examined bioplastics produced from *Chlorella pyrenoidosa.* The authors claimed that the bioplastics produced from *Chlorella pyrenoidosa* showed biodegradability in nature due to the presence of P(3HB) in the microalgae structure. P(3HB) production from the other strains of microalgae such as *C. reinhardtii, C. vulgaris,* and *C. fusca* has been recently reported [39,40].

Degradation rate, *Tg*, and *Tm* are affected by the type of monomers and by their composition [27,41]. While *Tm* and *Tg* of P(3HB) obtained from cyanobacteria have been reported in the ranges of 174–175 °C and 0.6–0.9 °C, respectively, P(3HB-co-3HV) had a *Tm* ranging between 148 and 168 °C and a *Tg* ranging from −5.5 to −2.2 °C [41]. Roja et al. [27] studied the thermal characterization of PHA polymer synthesized from marine algal species *Chlorella* sp., *Oscillatoria salina, Leptolyngbya valderiana*, and *Synechococcus elongates.* In this study, the PHAs were designated as PHA I, PHA II, PHA III, and PHA IV, respectively. FTIR testing revealed that PHAs contain functional groups such as stretching of –OH within carboxyl, C-H within methyl and methylene, C=O in ester, and C=O groups. The results of thermogravimetric analysis (TGA) and differential scanning calorimetry (DSC) showed that the degradation started at 217, 238, 261, and 249 °C for PHAs extracted from *Chlorella* sp., *Oscillatoria salina*, *Leptolyngbya valderiana*, and *Synechococcus elongates*, respectively. Thus, PHAs obtained from *Leptolyngbya valderiana* showed higher thermal stability compared to others. *Tg* and *Tm* of synthesized bioplastics were in the ranges of 4–10 °C and 79–116 °C, respectively. In a study conducted by Costa et al. [42], the PHAs extracted from *Spirulina* sp. LEB-18 and *Synechococcus subsalsus* were shown to be thermally stable below the temperatures of 250 and 287 °C, respectively (representing their thermal degradation). In the same study, the results of the DSC showed that the melting temperature occurred at around 171 and 173 °C for *Spirulina* sp. LEB-18 and *Synechococcus subsalsus,* respectively [42]. The band region around 1710–1750 cm^−1^ in FTIR spectra confirmed the presence of ester carbonyl group in the PHAs [42].

Bhati and Mallick [43] studied the efficiency of cyanobacteria *Nostoc muscorum* in the production of P(3HB) homopolymer and P(3HB-co-3HV) copolymer films compared with the PHAs produced from bacterial sources. They concluded that PHAs extracted from *Nostoc muscorum* have comparable thermal and mechanical properties with those of bacterial origin. In this study, P(3HB-co-3HV) copolymer films displayed a better performance than P(3HB) homopolymer. While the initial and maximum degradation temperatures of P(3HB) homopolymer from *N. muscorum* were 223 and 274 °C, the values registered for P(3HB-co-3HV) copolymer with 27 mol% HV units were 275 and 291 °C, respectively. PHAs produced from *N. muscorum* contained C-O and C/O stretching groups as well as stretching vibration of C\H bonds of methyl (CH_3_) and methylene (CH_2_) groups.

### 2.1. PHA-Based Blends and Composites

The blending of polymers is a simple approach that leads to improved polymeric materials. Many studies on PHA blends have been conducted to improve their properties and to reduce their production costs [44]. Cellulose, lignin, amylose, amylopectin, poly(lactic acid), and polycaprolactone are biodegradable polymers commonly used in blends with PHAs that result in improvements in the properties of PHAs [44]. Cellulose derivatives, e.g., ethyl cellulose, cellulose propionate, and cellulose acetate butyrate, were recognized as having high compatibility with PHAs, which make them attractive biomaterials for blending [45]. As previously discussed, most of the scl-PHAs exhibit high brittle behavior and crystallinity, and the latter property is incompatible with the high polymer flexibility required for application in the production of biomaterials. In this context, several authors and research groups have been studying different blends between PHAs and/or P(3HB) with cellulose derivatives to reduce crystallinity (e.g., [46]) or P(3HB) with an oligomeric mcl-PHA of microbial origin as plasticizer [47]. Blending PHAs with plasticizers is known as an important technique to increase the performance of most biobased plastics. Indeed, plasticization is achieved by their integration with raw polymers which in turn weakens the dispersion forces and hydrogen bonds [48]. Plasticizers, mineral fillers, and traditional impact modifiers are low-cost materials appropriate to reduce the brittleness and crystallinity of polymers and to improve flexibility and toughness of the final material as a result of lowering the glass transition temperature [48]. The ideal plasticizer needs to be biodegradable, nontoxic, and stable or nonvolatile [33]. Examples of common plasticizers are glycerol (considered as a conventional type of plasticizer for hydrophilic polymers [33], oxypropylated glycerin (or laprol), glycerol triacetate, polyethylene glycol, 4-nonylphenol, acetyl tributyl citrate, acetylsalicylic acid ester, salicylic ester, dioctyl phthalate, and dibutyl phthalate [49]. Behind the crystallinity and low cost, their physical and thermal properties are also of extreme importance. Bhatt et al. [50] showed that the thermal stability of mcl-PHAs could be enhanced by blending with natural and synthetic rubber. For instance, the thermal stability of poly(3-hydroxybutyrate-co-3-hydroxyvalerate) (P(3HB-co-3HV)) was improved after blending with poly(lactic acid), a chemically synthesized biodegradable polymer obtained from lactic acid. Despite each component exhibiting brittle behavior independently, their combination offers a clear advantage [51]. In general, by increasing the amount of PHAs in PHA/poly(lactic acid) blends, higher crystallinity and miscibility were observed [52]. The same behavior in the properties of polymers has been observed in lignin blends with PHAs [53]. It has been reported that the degradation rate of P(3HB) in P(3HB)/lignin blends can be reduced due to the formation of strong hydrogen bonds between lignin and P(3HB) [53]. The degradation rates of some immiscible binary blends such as P(3HB)/poly(propiolactone), P(3HB)/poly(ethylene adipate), and P(3HB)/P(3HB-co-3HV) were found to be higher than those of their pure counterparts due to their phase-separated structure [49].

According to [54], the compatibility of P(3HB) is enhanced by blending it with starch. The obtained results showed that the blends with different proportions of P(3HB) and starch showed a *Tg* in the range of 63.1–87.4 °C. The nature of all combinations was crystalline. Only the blend with 30% starch showed a significant increase in the tensile strength when compared with pure P(3HB). This type of blend has a lower cost than the ones based on P(3HB), since starch is a cheaper biopolymer that does not compromise the physical properties of the blend. A different study tested the blending of a starch derivative with P(3HB) [55]. Here, the blend was made with poly(vinyl acetate)-modified corn starch, and an improvement in compatibility and flexibility of blends was observed [55]. 

Other relevant properties are the toughness, elasticity, ductility and biological properties of the polymer, which can be improved when PHAs are blended with polycaprolactone, a biodegradable semicrystalline aliphatic polyester [56]. The authors reported that, despite the improved mechanical properties such as toughness, Young’s modulus, yield strength flexibility, and low degree of crystallinity [56] of P(3HB-co-3-HHx)/polycaprolactone blend (ratio 30:70), its behavior was not sufficient for tissue engineering applications. Considering the use of biopolymer blends using polycaprolactone to develop tissue engineering applications, other studies arose. Chiono and collaborators (2008) have produced hollow fibers using dry-jet wet spinning of P(3HB-co-3HV) and polycaprolactone solutions in chloroform [57]. According to their findings, the blends had a lower degree of surface and bulk porosity, and an increase in ductility was observed with the increase in polycaprolactone content in the P(3HB-co-3HV)/polycaprolactone blend [57].

Zhao et al. [58] showed that the mechanical and thermal properties of PHA were improved via adding 2 wt% ribbon-like hexagonal boron nitride nanoarchitectures as nanofillers. PHA/ribbon-like hexagonal boron nitride nanoarchitecture composites also showed enhancement in the ductility (strain at break), yield strength, and tensile strength by 52.3%, 49.4%, and 6.01%, respectively [58]. Table 1 presents the data from the literature contributing to the properties of various blends/composites of PHA.

### 2.2. Improvement of PHA Accumulation in Algae

To achieve an economically viable PHA production system from microalgae/cyanobacteria, the optimization of the PHA content is critical. Many researchers have attempted to increase PHA content either using genetic engineering approaches or through operational parameter optimization applied to the PHA production. The growth of microalgae can be influenced by several parameters such as light (quality and quantity), salinity, pH, temperature, and the amount and type of nutrients in the culture media [13]. However, saline stress has been shown to have no direct relationship with PHA production, although it can affect the increase in carotenoids and lipids [23]. Despite the importance of pH in increasing microalgal growth and lipid accumulation, the influence in PHA production has not been yet clearly studied. Several studies demonstrated that PHA content in microalgae can be increased by imposing thermal stress or nutrient limitation [72,73,74]. Since it reduces the formation of nitrogen compounds (like proteins), the limitation of nutrients such as nitrogen and phosphorus in the cultivation media of some microalgae species causes the increment in PHA content and other intracellular compounds (up to 20% in terms of dry cell weight) [74,75]. The high potential of cyanobacteria for PHA production often occurs under environmental stress such as nutrient-limited cultivation conditions [15,75]. Bhati and Mallick [43] investigated the effects of P and N deficiencies on P(3HB) accumulation in *Nostoc muscorum* Agardh, while analyzing the thermal and mechanical properties of P(3HB) and P(3HB-CO-3HV) copolymer films produced by the microorganism. An increase in the accumulation of P(3HB-CO-3HV) copolymer produced by *Nostoc muscorum* Agardh was obtained, this was up to 78% and 71% of dry cell weight under N and P deficiencies, respectively. Surprisingly, the thermal and mechanical properties of the polymer films produced by *Nostoc muscorum* were found to be comparable with the polymers produced by the cyanobacterium *Aulosira fertilissima* CCC 444 and the bacterium *Cupriavidus necator* and the commercial polypropylene, implying their capacity for large-scale production in the future [43].

Microalgae can be cultured under photoautotrophic (using light as a sole energy source to assimilate inorganic carbon sources), heterotrophic (microalgae obtain energy from organic substrates as carbon and energy sources in the dark), or mixotrophic conditions (microalgae use both light and organic carbon sources as energy sources) [76]. The results reported in the literature imply that the level of P(3HB) in cyanobacteria is less than 9% of their dry weight (*w*/*w* DW) under normal photoautotrophy [77,78]. However, P(3HB) accumulation in cyanobacteria can be increased remarkedly under nitrogen or phosphate limitation. Miyake et al. [79] reported that the P(3HB) level of *Synechococcus* sp. increased up to 27% (*w*/*w* DW) under deprivation of nitrogen, while in the study of Sharma et al. [80], the P(3HB) level of *Nostoc muscorum* reached 23% (*w*/*w* DW) under deprivation of phosphate. The filamentous *Calothrix scytonemicola* TISTR can produce 25.4% (*w*/*w* DW) P(3HB) using CO_2_ as a carbon source (photosynthetic system) under nitrogen deficiency [81]. Taepucharoen et al. [82] observed P(3HB-CO-3HV) accumulation enhanced (up to 42% *w*/*w* DW) in *Oscillatoria okeni* TISTR 8549 under heterotrophic conditions with nitrogen-sufficient conditions and in the presence of sodium acetate. Furthermore, Wu et al. (2002) showed that the P(3HB) accumulation in *Synechocystis* sp. PCC 6803 was up to 15.2% *w*/*w* under nitrogen-limited cultivation supplemented with sodium acetate, while for nitrogen-sufficient conditions, the P(3HB) content was up to 9.9% *w*/*w* [83]. Finally, *Aulosira fertilissima* CCC 444 was able to produce 77% (dry cell weight) of P(3HB) under phosphorus-limited conditions in the presence of fructose and valerate [84].

In another attempt to maximize the production, genetic engineering of microalgae/cyanobacteria has been applied, considering the increase in the conversion of CO_2_ into chemical compounds, particularly PHA/P(3HB). Several species such as *Synechocystis* sp. PCC 6803, *Synechococcus elongatus* PCC 7942, and *Synechococcus* sp. PCC 7002 can be used as naturally transformable candidates for genetic engineering applications [85]. Carpine et al. [86] investigated the overproduction of P(3HB) in *Synechocystis* sp. PCC6803 by overexpression of phosphoketolase combined with the double deletion of phosphotransacetylase and acetyl-CoA hydrolase under balanced growth conditions and using BG11 as the algal culture medium. The highest P(3HB) content (~12% (*w*/*w*) of the dry biomass weight) was obtained with a genetically engineered *Synechocystis* sp. PCC6803. To sum up, Table 2 shows the publications focusing on the PHA production through different mechanisms by algae.

## 3. Microalgal Proteins

Proteins are natural chains of 20 different amino acids connected by amide linkages forming polypeptide chains. The final properties of a protein are normally affected by the amino acid sequence. Intramolecular interactions, such as hydrogen bonding, electrostatic interactions, and hydrophobic interactions, are responsible for stabilizing the protein structure, including secondary, tertiary, and quaternary structures [100,101]. Plant proteins including corn zein, wheat gluten, peanut protein, and soy protein have been widely used for bioplastic production [22,48]. However, proteins seem to be an unsuitable feedstock for bioplastic production since they are known as a primary food source for human beings, which could cause a growing demand for using microalgal proteins as an alternative feedstock. Nevertheless, and in general, algae contain high protein content (10–47% cdw), which can become a value-added product when converted to bioplastic or thermoplastic blends [102]. A new biobased fiber with a diameter of 200 nm was produced from *Botryococcus braunii* residues containing 78% protein through an acidic-electrospinning process [103]. The mechanical properties and water vapor resistance of bioplastics can be positively affected by protein-rich biomass (*Chlorella sorokinan*) with polysaccharide addition [33].

To improve protein-based bioplastics’ functional properties, modifications of proteins including their denaturation by thermal or chemical treatments are necessary [22]. Wang et al. [102] developed algae bioplastics and their thermoplastic blends using protein modification of catfish algae and *Nannochloropsis* (protein-rich microalgae). After analysis of the thermal and dynamic mechanical properties of the materials developed, it was identified that the temperatures of degradation of *Nannochloropsis* and catfish algae were between 175 to 375 °C and peaked at 300 °C, which demonstrated the degradation of carbohydrates and proteins. The best processing temperature was found to be around 150 °C to yield the maximum denaturation, according to the DSC tests. Nevertheless, the *Nannochloropsis*-based bioplastics showed total flexibility, while the catfish algae bioplastic displayed a stiff behavior [102]. The modified thermoplastic derived from the blend of polyethylene (PE) or polypropylene (PP) with algae could induce a reasonable range of mechanical properties for targeted applications. Similarly, thermomechanical polymerization of proteins from *Spirulina* and *Chlorella* biomasses was applied to produce bioplastics and blends of microalgae biomass with PE+ in a study carried out by Zeller et al. [104]. They modified *Spirulina* and *Chlorella* through denaturation and thermoplastic blending. The thermal and mechanical characteristics of the polymers obtained were examined using DSC, dynamic mechanical analyzer (DMA), and tensile tests. Based on the results, denaturation peaks of *Spirulina* and *Chlorella* occurred at 100 and 110 °C, respectively. A viscoelastic behavior was observed in glycerol-plasticized *Spirulina* and *Chlorella* bioplastics (having 20% glycerol by weight) by DMA test. In this study, a low extension and high modulus were observed in both bioplastics. Zeller et al. [104] also suggested that both studied algae can be suitable alternatives for bioplastic and thermoplastic production. Although a better blend performance was attributed to *Spirulina*, *Chlorella* showed a better bioplastic behavior [104]. Moreover, the mechanical properties of algae bioplastics were observed to be comparable to soy protein isolate, feather meal, and duckweed.

The process of protein extraction from microalgae comprises breaking down polysaccharides to separate proteins or breaking down proteins into a more soluble form [105]. The extracted proteins are then dissolved out of the fibrous structure, followed by separation and drying. There are several methods that can be employed for protein extraction. Generally, these extraction methods can be divided into chemical, physical, and enzyme-assisted extraction; ultrafiltration; and diafiltration. Alkali or acidic solution (sodium hydroxide, hydrochloric acid, polyethylene glycol, potassium carbonate, and N-acetyl-cysteine) is required for extraction of the protein with a chemical method via disrupting cell wall structure [105]. In physical extraction processes, mechanical forces, including shear force (such as grinding and homogenizing) and osmotic pressure (such as immersion in water), are dominant actions for separating the water-soluble protein from the fiber [106]. Enzyme-assisted extractions are another strategy for protein extraction and are associated with higher yields, up to 87%, in comparison to the other conventional methods, such as the physical method with yields of up to 40% [107]. However, this method is not commercially feasible because it demands high concentrations of enzymes [108]. In fact, assisted extraction is a modification of conventional methods by additional techniques to improve the protein extraction process. Ultrasound, microwave-assisted method [105,109], subcritical water or supercritical CO_2_ [110], membrane technology, and pulsed electric field [111] are examples of assisted extraction processes. For instance, protein extraction yield has been increased by 13% and 27% (protein extracted as a percentage of protein present in the biomass) from *Spirulina* and *Chlorella*, respectively, through pulsed electric field combined with conventional method [112]. The yield and quality of the extracted protein can be optimized using concurrent ultrafiltration and diafiltration (UF/DF) processes [111]. Different processes have been examined in the literature for extracting proteins from algae such as deionized water, Tris-HCl buffer, aqueous polymer two-phase system (PEG/K_2_CO_3_), and polysaccharidases [113]. The highest level of protein extraction was reached following a first extraction with deionized water and a second extraction with NaOH (0.1 M) [113].

### 3.1. Protein-Based Blends and Composites

Bioplastics can be produced using *Spirulina* sp. (the cyanobacteria species most applied commercially nowadays) and its proteins. Moreover, an innovative blending system has been proposed by Wang [114] to improve the performance of bioplastics conventionally produced from 100% *Spirulina* biomass. The system consists of plasticization using ethylene glycol (EG), blending with ultrahigh-molecular-weight polyethylene (UHMW-PE), and compatibilization using polyethylene-graft-maleic anhydride (PE-g-MA) with the *Spirulina*. Bicinchoninic acid (BCA) test assay and sodium dodecyl sulfate–polyacrylamide gel electrophoresis (SDS-PAGE) can be used to measure the protein content and approximate molecular weight, respectively [114].

### 3.2. Improvement of Protein Content in Microalgae

Cultivation strategies can influence the productivity of proteins in microalgae [115]. The protein content of microalgae can be increased when cultured under nonstress conditions, while different conditions such as halostress [115] and nutrient starvation [116] result in an accumulation of carbon-rich molecules (e.g., lipids and carbohydrates). Cultivations of *Scenedesmus* using semicontinuous strategies yielded biomasses with twice the protein content in a study conducted by Rocha et al. [115] when compared to cultivations performed in a single batch. Nitrogen is another factor affecting the production of proteins in microalgae [117]. The use of low-cost nitrogen sources such as agricultural fertilizers and urea is recommended for the nutrition of some microalgae strains [118,119]. Light quality and intensity, nutrients, temperature, halostress, carbon supply and source, and climate conditions are some other parameters able to improve the protein productivity in microalgae [120,121,122,123]. 

As in the case of PHA/PHB accumulation, the protein content and quality in microalgae may also be improved by genetic engineering and synthetic biology tools, as recently reported in [124]. The authors concluded that protein content and biomass production were improved by 31.8% and 11.6%, respectively, after modification of *C. pyrenoidosa* (K05) through irradiation-mediated mutagenesis.

## 4. Microalgal Starch

Starch is an abundant, digestible, and biodegradable polysaccharide that can be used in food, textile, paper, and packaging industries [125]. It is a common polysaccharide comprising D-glucose monomers joined by glycosidic bonds. Starch possesses different proportions of amylose (10–20%) and amylopectin (80–90%), and its properties mainly depend on the different proportions. An increased proportion of amylopectin results in the increase in crystallinity of starch, while an increment in amylose leads to higher tensile strength, higher Young’s modulus, and lower elongation at break, all basic requirements for the production of bioplastics [33,126]. This being said, three approaches can be adopted for using starch in polymeric materials [127]:

(1) Indirectly converting starch into the monomers, which are used in the synthesis of polymers such as poly(lactic acid) from lactic acid, polyethylene from ethylene prepared by ethanol dehydration, or even PHAs.
(2) Using starch as a raw material to produce low-molecular-weight hydroxylated compounds. Dextrins and glycolized products are two examples of polymers used in polyurethane formulations.
(3) Using starch as a filler in other plastics or as thermoplastic starch.

Among the above-mentioned approaches, the third one is considered the most economical and simplest method to produce biopolymers. In this method, the macromolecular nature of starch remains unmodified. In nature, starch appears in semicrystalline granules, whose shape and size depend on the source of starch. These granules comprise minor components such as lipids, proteins, and inorganic compounds [128]. Native starch molecules are insoluble in cold water and resemble spherulites with alternating amorphous and crystalline (or semicrystalline) lamellae. Starch can be also modified by continuous physical processes, of which extrusion is a good example [128].

Microalgae produce small starch granules (narrow size distribution of 0.5–2.1 µm) and are considered as new feedstocks for starch-based bioplastic production [129]. Microalgae are sources of carbohydrates, namely starch and cellulose, with the advantage of no lignin present [130]. The development of starch-based bioplastics using microalgal starch was investigated by Mathiot et al. [130]. Maximum starch production was observed in *Chlamydomonas reinhardtii* 11-32A strain with up to 49% *w*/*w* after 20 days of sulfur deprivation (under autotrophic conditions), corresponding to a concentration of 5.07 g·L^−1^ in flasks. Twin-screw extrusion was used to directly promote the successful plasticization of starch-enriched microalgal biomass with glycerol. In another study, the appropriateness of starch from marine microalgae *Klebsormidium flaccidum* to produce a starch-based bioplastic was assessed together with starch content in algal biomass. Starch characteristics, such as the size of its granules, amylose/amylopectin content, swelling power, solubility, and turbidity were studied to ensure the suitability of starch produced from marine microalgae in bioplastic formulations [131]. The results were compared with corn starch, widely used in the production of bioplastic, and demonstrated the high viability of the approach using starch from microalgae [131].

### 4.1. Starch-Based Blends and Composites

Starch has been considered as the major component in polymer blends due to the outstanding properties it promotes. Although the starch-based polymers are characterized by low mechanical and barrier properties, their combination with other polymers shows promising features such as safety, biodegradability, and sustainability [132]. Poly(lactic acid), for example, is a polymer with high mechanical properties, although has high cost and limited availability. However, a blend of starch and poly(lactic acid) can show both improvements in mechanical properties and lower production costs, which has been justifying the recent investigation on their use in the food packaging manufacturing industry [127,132]. Granular starch can be used as a cost-effective filler for the polymer when mixed with molten thermoplastic. To avoid gelatinization of starch, the mixing process should be conducted below the thermal degradation temperature of starch. Despite the advantages of using starch in the form of a blend, its use in high contents in the mixture can be associated with the reduction of the blend’s mechanical properties [133]. In the case of plasticizing starch granules under heating, the continuous phase can be raised in the form of a viscous melt following injection molding and extrusion techniques [134]. Plasticized starch is known as thermoplastic starch and is used in polymer blends. It possesses low mechanical strength and high water vapor permeability (WVP) [126]. The examples of plasticizers tested with thermoplastic starch are glycerol, water, urea, formamide, ethylenebisformamide, sorbitol, citric acid, N-(2-hydroxyethyl)formamide, and amino acids. Despite the high efficiency of water as a plasticizer, again glycerol is the plasticizer most used in the preparation of thermoplastic starch thanks to its high boiling point, availability, and low cost [135]. Thermoplastic starch can be blended with biodegradable polymers such as polycaprolactone, poly(lactic acid), and P(3HB-CO-3HV) [136,137] in order to produce new value-added products with improved mechanical, barrier, and water-resistance properties [138,139]. The mechanical properties of thermoplastic starch/polycaprolactone blends strongly depend on the plasticizer content, the lower level of which allows obtaining a blend with lower elastic modulus but improved impact strength. However, the addition of polycaprolactone in a rubbery thermoplastic starch led to an inverse trend [140]. Moreover, the melting point of the starch–polycaprolactone blend has been marked as low as 60 °C, which limits its application [141]. On the other hand, blending thermoplastic starch with nanocomposites or clay matrices has been found to improve the mechanical properties and thermal barriers of the blended materials [133].

### 4.2. Improvement of Starch Content in Microalgae

#### 4.2.1. Nitrogen, Phosphorous, and Sulfur Limitation

It has been established that nutrient-starved conditions are necessary for the optimum growth of microalgae. Consequently, the use of nutrient-limited media resulting in the accumulation of non-nitrogenous compounds is an approach commonly applied [130,142,143]. Dammak et al. [142] investigated the effect of limiting NaNO_3_, NaH_2_PO_4_, metals, and vitamins on the biochemical compositions of *Tetraselmis* sp. The maximum content of starch (42% DW) in *Tetraselmis* sp. was obtained under nitrate, phosphate, metal, and vitamin limitations. Dragone et al. [144] increased the microalgal starch content by applying a two-step approach: in the first step, the cells were grown in a nitrogen- and iron-sufficient medium; in the second step, the biomass was subjected to nutrient limitation, allowing the accumulation of starch content of more than 40% in *Chlorella vulgaris* [144]. The same rationale was followed by Hing et al. [143], where phosphorus and nitrogen limitations were successfully applied to maximize the production of carbohydrates, particularly almost doubling the production of starch, in *Arthrospira platensis* and [145] in *Chlorella zofingiensis* by nitrogen starvation (maximum content of starch 66.7% by nitrogen starvation). Besides the limitation of nitrogen and iron, sulfur starvation was also considered and identified as the most appropriate treatment for the scaled-up production of starch-enriched biomass [129]. The effects of nutrient limitation (nitrogen, sulfur, phosphorus) and the use of 1 mg/L of cycloheximide, as a specific inhibitor of cytoplasmic protein synthesis, on the starch content in *Chlorella* were tested by Brányiková et al. [129]. In laboratory-scale photobioreactors, the starch content increased up to about 60% DW under cycloheximide inhibition or sulfur limitation. In the case of phosphorus or nitrogen starvation, cellular starch content increased up to 55% or 38% DW, respectively. However, algal growth and starch accumulation decreased after about 20 h. Investigation in outdoor pilot-scale reactors showed that starch content in *Chlorella* reached 50% DW under sulfur-limited conditions. In summary, it is believed that the limitation of macronutrients, namely nitrogen and phosphorus, leads to an improvement in the production of polysaccharides and lipids, while reducing the protein content [142,146,147,148]. Starch productivity of a microalga (*Chlorococcum* sp. TISTR 8583, which was cultivated on BG-11 medium), reached 34.02% total sugars under nitrogen limitation compared to 22.57% on nitrogen-supplemented (NS) media only [149].

#### 4.2.2. Temperature and Irradiance

Temperature and light intensity are also important factors greatly influencing the carbohydrate content and the algal growth rate [150]. Although the effect of temperature on enzymes involved in carbohydrate synthesis such as starch synthase and sucrose synthase has been revealed in the study carried out by González-Fernández and Ballesteros [151], its effect on the carbohydrate accumulation in microalgae remains unclear [152]. As an example, the carbohydrate content in *Chlorella vulgaris* SO-26 decreased from 70 to 50% when the temperature rose from 5 to 20 °C [153]. The effect of temperature on the production of starch in *Chlamydomonas reinhardtii* was studied by Mathiot et al. [130]. They found that an increase in temperature up to 39 °C could result in a complete block of nuclear and cellular division accompanied by an increased accumulation of starch. The supraoptimal temperature in this study also caused a reduction in time needed to reach the maximum starch content, 1–2 days compared with 5 days for the culture at 30 °C [130].

The growth of carbohydrates in the microalgae is highly influenced by the range of the light intensity. Higher light intensity leads to the higher production of polysaccharides in the microalga [144]. In a study by Markou et al. [154], cultivating *Arthrospira platensis* under the irradiance range of 24–60 μmol_photons_·m^−2^·s^−1^ had no effect on carbohydrate content, despite its advantageous effect on biomass production. For the same reason, Samiee-zafarghandi et al. [155] investigated the effect of nutrient limitation (10–200 mg_NaNO3_·L^−1^ and 10–70 mg K_2_HPO_4_·L^−1^) and different light intensities (60–450 μmol_photons_·m^−2^·s^−1^) on the carbohydrate content of *Chlorella* sp. They found that the highest carbohydrate content, 60.9%, was obtained in nutrient limitation with 10 mg·L^−1^ of K_2_HPO_4_ and 105 mg·L^−1^ of NaNO_3_, as well as a light intensity of 255 μmol_photons_·m^−2^·s^−1^. The best range of light intensity for microalgae cultivation was found to be 180–540 μmol_photons_·m^−2^·s^−1^ in the study of Ho et al. [156]. They found that the highest carbohydrate content can be achieved at the light intensity of 400 μmol_photons_·m^−2^·s^−1^.

#### 4.2.3. Inorganic Carbon

Inorganic carbon, including CO_2_ and HCO_3_^-^ ions, has been recognized as an essential element for the accumulation of carbohydrates and synthesis of lipids in microalgae [157]. Using sodium bicarbonate at certain concentrations, a more stable form of CO_2_ in the form of HCO^−^_3_ ions has been proved as the most suitable approach to improve carbohydrate accumulation and biomass productivity in microalgae [158], although the increase elevated the pH of the media. As another example is the study conducted by Pancha et al. [159], where the optimum concentration of sodium bicarbonate was found to be 0.6 g·L^−1^ for the growth of *Scenedesmus* sp. CCNM 1077, resulting in an increase in carbohydrate synthesis up to 26%. They also observed a further improvement in carbohydrate content up to 54.03% under nitrogen starvation coupled with bicarbonate supplementation conditions [159]. The effectiveness of directly using CO_2_ and other carbon sources such as pentose to improve the carbohydrate content can be found in several other published works [160,161,162]. Literature reports that both the source and concentration of CO_2_ are important. Astri Rinanti [163], for example, studied the impacts of different CO_2_ concentrations, 5%, 10%, 15%, and 18% (*v*/*v*), on the carbohydrate and lipid production in three species of microalgae, *Chlorella vulgaris, Scenedesmus obliquus*, and *Ankistrodesmus* sp., grown under controlled conditions in a photobioreactor. Their results showed that the production of carbohydrates and lipids was increased by the addition of 10% (*v*/*v*) of pure CO_2_ at a flow rate of 5 L·min^−1^ into the photobioreactor. Other studies reported the effect of CO_2_ concentration, namely [160], which indicated carbohydrate content of 58.45% in *Coelastrum* sp. SM cultivated in dairy wastewater supplemented with 12% CO_2_, and the study of de Freitas et al. [162], which reported an increase in carbohydrate content of *Chlorella minutissima* pentoses from 32.5 to 56.8% with 5% pentose supplementation conditions. Many researchers have improved microalgal carbohydrate content by genetic engineering methods such as overexpressing the enzymes involved in the starch synthesis or via inactivating starch catabolic enzymes [164,165].

## 5. Microalgal Cellulose

Cellulose is a biocompatible, renewable, biodegradable, and natural polymer consisting of a linear chain polysaccharide of repeating D-glucose units joined by β(1 → 4) glycoside linkage [166,167]. Cellulose is a rigid, fibrous, highly crystalline, and water-insoluble polysaccharide, often used as a filler for reinforcement of bioplastics [166]. Many algal taxa contain cellulose and hemicellulose. In higher plants and algae, cellulose is surrounded by a matrix containing hemicellulose, pectin, and lignin and supports the cell wall [168]. Cellulose can be isolated from several parts of plants and algal cell walls [166]. Due to limited information about the cell wall ultrastructure of many species of algae, finding a relevant cellulose extraction method is hindered [169]. The composition of the microalgal cell wall may vary between species. The cell wall of algae belonging to the Prasinophytina and Chlorodendrophyceae is composed of 2-keto-sugar acids 3-deoxy-manno-2-octolusonic acid, 3-deoxy-5-O-methyl-manno-2-octolusonic acid, and 3-deoxylyxo-2-heptulosaric acid. Unicellular algae related to Trebouxiophyceae and Chlorophyceae are characterized by cell walls consisting of mannans, glucans, arabinogalactans, algaenans, and less frequently, chitin-like polysaccharides [170]. *Chlorella sorokiniana*, *Chlorella vulgaris*, and *Chlorella kessleri* are classified in the glucosamine-rich rigid cell wall group. Rhamnose and galactose are the main sugars in the hemicellulose fraction of the glucosamine-rich rigid cell wall [171]. The cell wall of *Neochloris oleoabundans* contains about 24.3% carbohydrates, 31.5% proteins, 22.2% lipids, and 7.8% inorganic material [171].

The cellulose content in filamentous green algae has been found to be around 20–45% [172,173]. Cellulose content in *Lyngbya* sp. has been reported to be about 14–17% [174]. High crystallinity of about 95% was observed in the cellulose extracted from *Valonia* and *Cladophora* species belonging to Cladophorales and Siphonocladales families [175]. In the research of Constante et al. [176], the tensile strength and modulus of cellulose extracted from *Lyngbya* sp. were 215 MPa and 24 GPa, respectively. Mihranyan [172] showed that cellulose derived from *Cladophora* sp. can be used to reinforce materials applied in the preparation of bioplastics. Several drawbacks are associated with natural cellulose fibers, namely their poor thermal stability, noncompatibility with hydrophobic polymers, and moisture absorption [166]. These problems can be solved by using various forms of cellulose such as nanofibers, cellulose nanofibrils (CNFs), and microcrystalline cellulose. Furthermore, composites with improved properties can be produced through the combination of readily available cellulose with adequate polymers [152,155,166]. The high mechanical property of microcrystalline cellulose and its high crystallinity makes it a suitable candidate as a reinforcing material [177] and for improving the properties of biopolymers such as poly(vinyl alcohol) [178] and poly(lactic acid) [179]. Cellulose nanofibers exhibit excellent mechanical strength, high flexibility, biodegradability, chirality, thermostability, and low thermal expansion [166]. For this reason, cellulose nanofibers extracted from algae can be applied as a reinforcing agent in polyurethane foams, offering better tensile strength, elastic modulus, biodegradability, and thermal resistance [166]. CNFs are also characterized by high tensile modulus, light weight, surface activity, and biocompatibility, being suitable to be used as a filler in the field of composites (packaging and paper industry), a stabilizer in emulsions, a conducting sheet in electronics, or a biomaterial in a medical application [180]. Nevertheless, their application as a reinforcing filler has been limited due to high energy and time requirements. Large amounts of cellulose nanofibrils could be easily isolated from *Nannochloropsis* species by a simplified purification process, since their cell wall consists essentially of cellulose (about 75%) without hemicellulose and lignin [181]. Lee et al. [180] studied a new method to obtain cellulose nanofibrils from *Nannochloropsis*; their results show the advantages of using microalgae as a source of cellulose nanofibrils, as this reduces the demands of energy and time required for mechanical treatments. These advantages may result from their small size, rapid growth rate, and high productivity. The tensile strength of cellulose nanofibrils obtained from *Nannochloropsis oceanica* was about 3–4 GPa, which is similar to or even higher than that of other cellulose nanofibrils and general reinforcements currently applied [180].

The use of algal cellulose nanocrystals (CNCs) from red algae waste and CNFs from *Nannochloropsis* sp. as a reinforcing filler to produce biocomposite films has been recently studied [180,182]. Despite the interesting properties of microalgal nanocellulose such as CNCs and CNFs, their application is limited mostly to the production of reinforcing filler rather than bioplastics. CNF-based films exhibited barrier properties comparable to those of conventional petroleum plastics [169]. Furthermore, they are suggested as a candidate in packaging applications since they have better properties than films from CNCs [183].

### Cellulose-Based Blends and Composites

Cellulose-based composites, blends, and nanocomposites have been used in many applications such as composite materials, tissue-engineered cartilage scaffolds, wound dressings, artificial skin, dental implants, catheter covering dressings, dialysis membranes, coatings for cardiovascular stents, cranial stents, membranes for tissue-guided regeneration, controlled drug release carriers, vascular prosthetic devices and artificial blood vessels, optoelectronic materials, optical coating, packaging materials, and electrical materials for electrical applications [184]. Cellulose-based nanocomposites are normally formed by adding cellulose nanoscale fillers to the polymer matrices, thus leading to an enhanced mechanical reinforcement. Nyström et al. [185] produced a *Cladophora* cellulose nanofiber composite by coating the fibers with polypyrrole and polyaniline polymers. According to their results, conductive properties are associated with composites formed by polypyrrole/*Cladophora* cellulose nanofibers, allowing their potential use in ion exchange resins and paper-based battery fabrication. Microalgae can be used to produce a small-diameter cellulose fiber (from micrometer to nanometer scale) with a better reinforcement performance via electrospinning technique [12,152]. Sustainable nanocelluloses such as those composed of polycaprolactone and *Spirulina* sp. [186] and those composed of 10% microalgal biomass (*Scenedesmus almeriensis*) coupled with poly(ethylene oxide) [187] are the successful examples produced using the electrospinning technique. A poly(ethylene oxide)–*Spirulina* sp. mixture was used by de Morais et al. [188] to produce nanocellulose biocomposites with a diameter of 110 nm to be used in tissue engineering. Finally, very few works have focused on the effect of the culture conditions on the content of cellulose produced. The main conclusions from the few works available relate to the effect of light and the type of production (i.e., photoautotrophic, heterotrophic, and mixotrophic growth) [189].

## 6. Conclusions, Future Perspectives, and Personal Reflections

The concerns demonstrated by society and governments have been crucial to include in all stakeholders’ conversations on the impact of plastics. Moreover, the need to create a circular economy for these products [190] applied to all commercial sectors, including food, health, cosmetic, and nutraceutical industries and services, is a real priority. It is recurrently argued that bioplastics, as part of the bioeconomy, are a perfect image of circularity. They not only regenerate CO_2_ but use renewable raw materials, as reviewed in this work and others, to make more sustainable daily products.

The efforts being made by governments and official institutions demonstrate the urgency of overcoming the problem of non-biodegradable plastics. One of the most recent initiatives of the European Commission (EC) clearly shows the need to change habits and consumption through the EU Green Deal. As the EC explains, “*The European Green Deal provides an action plan to (i) boost the efficient use of resources by moving to a clean, circular economy and restore biodiversity and cut pollution*” [191]. To reach this target, the EU Green Deal action indicates that initiatives will be required in all sectors of the economy. These, in their opinion, include production (*to invest in environmentally friendly technologies, to support industry to innovate*), services (*rolling out cleaner, cheaper, and healthier forms of private and public transport*), energy (by *decarbonizing the energy sector*), habitation (*ensuring the energy-efficiency of buildings*), and environment (*working with international partners to improve global environmental standards*, *avoid pollution, and protect the ecosystems*). In this context, the problem of using petrol-based plastics as a significant source of (terrestrial and marine) pollution is not new for academia, governments, or society. The numbers are impressive, since more than 14 million tons of plastics are polluting the oceans and around 91% of the plastic used worldwide is not recycled [192]. The development of bioplastics has been investigated and discussed by academia, as reviewed in this work. Both producers and industrial end-users of plastics are addressing the problem and the potential solutions by participating in conferences (e.g., World Economic Forum Annual Meeting [193]), debates [194], and round tables [195] and by financing/participating in projects aiming to solve the problems of their own companies (e.g., Novamont or Saponia through the European project CIRC-PACK [196] or Unilever [197]). It is mainly under the context of food packaging that the study of bioplastics has been more intensive. The most recent data indicate that the global market for bioplastics was defined as being around 2.11 million tons in 2018 and is estimated to reach 2.62 million tons by 2023 [198]. Nowadays, the main biobased plastics commercially exploited include poly(lactic acid); PHAs; and starch-, cellulose-, and protein-based plastics, which, at least in part, justifies the high interest of authors in these materials as reviewed here.

Despite the efforts made by the different researchers, in our opinion, much more needs to be done to overcome this problem and to bring the use of bioplastics to a daily base regime. As demonstrated in this work, the use of microalgae and cyanobacteria to produce the starting materials for the more efficient production of bioplastics is an important issue to consider. The advantages are well explored; microalgae and cyanobacteria are not competing for arable land and are able to produce significant quantities of proteins, starch, cellulose, and PHAs. However, the lack of efficiency and sustainability in the production processes, as reviewed, needs further attention. Nevertheless, it is required to demonstrate the need for fundamental research contemplating the appropriate selection of the algal strain and culture conditions. Moreover, deeper studies are required, including the effective manipulation of microalgal metabolism and investigation of possible genetic modifications required to further enhance the efficiency and performance of algal bioplastic starting materials. Nowadays, bioplastics already play an important role in the packaging, agriculture, gastronomy, consumer electronics, and automotive fields. However, they are still a very small part (around 1%) of the whole picture of the plastic business. The data available in this work on the use of different compounds as starting materials to produce bioplastics and others recently published approaching the different facets of the same issue, the efficient production (this work and others [14,199]), use [199], and disposal of bioplastics [199], highlight bioplastics as part of the solution to overcome the problem imposed by the excessive use of petrol-based plastics. Although we have not reviewed the applications of bioplastics and the disposal strategies for bioplastics in this work, since it was previously done by other researchers [14,182], we could not neglect the importance of both issues. It is true that bioplastics, being biodegradable, give a better answer to this problem; however, we should not forget that this is just an additional property of the material and that efficient and effective strategies are still needed for its disposal and end-of-life management. Recently, a revision of the European Union (EU) waste package was processed, which is another key issue of the Circular Economy Action Plan. The revised EU waste legislation included a revision of the Waste Framework Directive. Indeed, since 2018, this revised Waste Framework Directive has allowed biodegradable and compostable packaging to be collected simultaneously with the biowaste and recycled in industrial composting and anaerobic digestion. By 2023, separate collection of biowaste is set to be mandatory throughout Europe. However, despite all the efforts, the Directive still lacks specific legislative measures stimulating the use of bioplastics and improving the market conditions for bioplastic products and derivatives.

## Figures and Tables

**Table 1:** Properties of various blends/composites of PHA.

Polymer Blend (mol·mol^−1^)	Melting Temperature (°C)	Glass Transition Temperature (°C)	Tensile Strength (MPa)	Young’s Modulus (MPa)	Elongation at Break (%)	Applications	Ref.
P(3HB)/starch (70/30)	165.4 to 167.9	165.4 to 167.9	4.99 to 19.7	578 to 1716	3.5 to 9.8	Coating materials, cardboard for food package	[54]
P(3HB)/PIP-g-PVAc (80/20)	175	6	14.3	711	13		[59]
P(3HB)/starch acetate	171.0 to 175.9	8.6 to 9.9	-	-	-	-	[60]
P(3HB)/ethyl cellulose	175.3 to 177.0	44.6 to 56.1, annealed samples	-	-	-	-	[60]
P(3HB)/cellulose acetate butyrate	178.5 to 189.5	6.3 to 12.5	13.3 to 29.3	592.4 to 2288.3	2.2 to 7.3	-	[61,62]
P(3HB)/lignin	152 to 174	7.0 to 43.0	-	-	-	-	[53]
P(3HB)/P(3HHx)	Approximately 152 to 165	Approximately 0.8 to 5.0	-	500 to 1210	-	Scaffolds for tissue engineering with improved biocompatibility	[63,64]
P(3HB-co-4HB)/PLA stereocomplex (SC)	PLA SC: 218	P3/4HB: −12.5	4.2 to 6.6	30.8 to 46.7	362.7 to 949.0	Enhanced processability and enzymatic hydrolysis rates	[65]
P(3HB-co-3HHx)/PCL	P(3HB-co-3HHx): 95.4 PCL: 61.1	-	-	190.9 to 324.6	-	Improved cell adhesion and proliferation for musculoskeletal tissue engineering	[56,66]
P(3HB-co-3HV)/PCL	PCL: 57.0 to 57.5 P(3HB-co-3HV): 137 to 152.4	P(3HB-co-3HV): 1.3	-	170 to 1200	8.0 to 25	Hollow fibers and tubular scaffold in tissue engineering	[57,67]
P(3HB)/PLC (77/23)	60 to 168	−60 to 4	21	730	9	-	[68]
P(3HB)/P(3HO) (75/25)	172	−35	6.2	730	30	-	[69]
P(3HB)/P(3HB-co-3HV) (25/75)	152 to 163	-	2	150	7	Electrospun fiber mats of poly(3-hydroxybutyrate), poly(3-hydroxybutyrate-co-3-hydroxyvalerate), and their blends	[70]
P(3HB-co-3HV)/a-P(3HB) (50/50)	133	2	7	240	33	-	[71]

**Table 2:** Production of PHAs by algae through different mechanisms.

Produced Polymer and Operational Conditions	Algae Used	Polymer (%Dry Cell Weight)	Ref.
P(3HB) production using CO_2_ as carbon source (photosynthetic system)	*Synechocystis* cf. *salina*	7.5	[87]
P(3HB-CO-3HV) production under nitrogen deprivation	*Oscillatoria okeni* TISTR 8549	14.4	[82]
P(3HB-CO-3HV) production under nitrogen deprivation and dark condition	*Oscillatoria okeni* TISTR 8549	42.8	[82]
P(3HB) production under phosphate-starved medium + 1% (*w*/*w*) glucose + 1% (*w*/*w*) acetate with aeration and CO_2_ addition	*Nostoc muscorum*	21.5	[88]
P(3HB) production using CO_2_ as carbon source (photosynthetic system) under nitrogen deficiency	*Calothrix scytonemicola* TISTR 8095	25.4	[81]
PHA production using BG11 as culture medium	*Synechocystis salina*	5.5–6.6%	[26]
PHA production under phosphorus and nitrogen deficiency	*Synechococcus elongates*	17.15	[89]
PHA production under phosphorus deficiency	*Synechococcus elongates*	7.02	[89]
PHA production using wastewater as culture medium	Microalgae consortium	43	[90]
PHA production under nitrogen deficiency	*Synechococcus subsalsus*	16	[42]
PHA production under nitrogen deficiency	*Spirulina* sp. LEB-18	12	[42]
P(3HB) production using 0.11% acetate and 0.08% propionate at pH 8.1 and an incubation period of 16 days	*Nostoc muscorum*	31	[91]
P(3HB) production using 0.2% acetate and 0.4% propionate, incubation period of 14 days at pH 8.5	*Nostoc muscorum*	28.2	[91]
P(3HB) production under phosphorus limitation	* Spirulina maxima *	1.2	[92]
P(3HB) production under phosphorus limitation, supplemented with acetate (dark incubation for 7 days)	*Nostoc muscorum*	35	[80]
P(3HB-CO-3HV) production under phosphate deficiency conditions	*Nostoc muscorum* Agardh	71	[43]
P(3HB-CO-3HV) production under nitrogen deficiency conditions	*Nostoc muscorum* Agardh	78	[43]
Production of P(3HB) using CO_2_/acetate as carbon source	*Spirulina plantesis*	10	[93]
Production of P(3HB) under phosphate deficiency with gas-exchange limitation (GEL) conditions and using fructose/acetate as carbon source	*Synechocystis* sp. PCC6803	38	[91]
Production of P(3HB) using 0.2% acetate/dark incubation for 7 days	*Nostoc muscorum*	35	[80]
Production of P(3HB) under phosphate limited conditions and permanent illumination	Mixed cyanobacterial culture: *Aphanocapsa* sp. and cf. *Chroococcidiopsis* sp.	838 mgL^−1^	[75]
Production of P(3HB) under nitrogen-limited conditions,	*Synechocystis* sp. UNIWG and *Synechocystis* sp. PCC 6803	14	[15]
Production of P(3HB) in genetically engineered systems	Incorporation of phbB and phbC genes from *R. eutropha* into *C. reinhardti*	-	[94]
Production of PHB in genetically engineered systems	Incorporation of full PHB pathway from *R. eutropha H16* into *P. tricornutum*.	10.6	[95]
PHB production in genetically engineered systems under nitrogen-limited conditions	*Synechocystis* sp. (genetically modified with overexpressing pha genes)	35	[96]
Improvement in PHA production after UV light exposure	*Synechocystis* sp. PCC6714	37	[97]
PHB production under nitrogen deficiency and using acetate as carbon source	*Synechococcus* sp. PCC7942	26	[98]
PHB production under 0.26% citrate, 0.28% acetate, and 5.58 mg L^−1^ K_2_HPO_4_ (incubation period of 5 days)	*Aulosira fertilissima* CCC 444	85	[41]
PHB production under nitrogen deficiency	* Spirulina platensis *	10	[99]
PHB production under nitrogen deficiency	* Synechocystis * sp. UNIWG	14	[99]
P(3HB-CO-3HV) production under phosphorus deficiency and under 0.5% fructose + 0.4% valerate	* Aulosira fertilissima * CCC 444	77	[84]
P(3HB-CO-3HV) production under nitrogen deficiency with acetate supplementation under dark condition	*Oscillatoria okeni TISTR 8549*	42	[82]
